# Competency of Infection Control Nurses and Associated Factors: A Cross‐Sectional Study

**DOI:** 10.1155/jonm/6935833

**Published:** 2026-04-29

**Authors:** Wan Li, Pan Lin, Qiuhong Yang, Haifan Yang, Xin Tan, Xinyu Feng, Hui Luo, Weijuan Li, Yinglan Li, Ying Zhang, Lingyun Tian

**Affiliations:** ^1^ Department of Clinical Nursing Teaching and Research, Xiangya Hospital, Central South University, Changsha, China, csu.edu.cn; ^2^ National Clinical Research Center for Geriatric Disorders (Xiangya Hospital), Changsha, China; ^3^ Clinical Nursing Research Center of Central South University, Changsha, China, csu.edu.cn; ^4^ Department of Nursing, The Second Affiliated Hospital of Zhejiang University School of Medicine, Hangzhou, China, z2hospital.com; ^5^ Department of Lymphoma and Hematology, Hunan Cancer Hospital, Changsha, China, hnzlyy.com; ^6^ School of Nursing, The Hong Kong Polytechnic University, Hong Kong, China, polyu.edu.hk; ^7^ Department of Health Management Center, Division of Life Sciences and Medicine, The First Affiliated Hospital of USTC, University of Science and Technology of China, Hefei, 230001, Anhui, China, ustc.edu.cn; ^8^ Department of Nursing, Division of Life Sciences and Medicine, The First Affiliated Hospital of USTC, University of Science and Technology of China, Hefei, 230001, Anhui, China, ustc.edu.cn; ^9^ Division of Life Sciences and Medicine, Institute of Public Health Sciences, University of Science and Technology of China, Hefei, 230001, Anhui, China, ustc.edu.cn

**Keywords:** competency, infection management nurses, influencing factors, nosocomial infection, status

## Abstract

**Background:**

Nosocomial infections (NIs) are a global concern, straining healthcare resources and patient safety. Infection control nurses (ICNs) play a crucial role in NI management. However, comprehensive surveys assessing ICNs’ competency are lacking. This study aimed to assess the competency of ICNs in China and identify influencing factors.

**Methods:**

A cross‐sectional survey was conducted between December 2021 and January 2022 across 14 cities in Hunan Province, China. Using convenience sampling, 1010 ICNs completed an online self‐designed questionnaire comprising 69 items. Multiple linear regression analyses were performed to identify factors associated with ICNs’ competency.

**Results:**

A total of 946 valid questionnaires were collected. The overall competency score of ICNs was 272 points (P25–P75: 253–300). Among the six primary competency domains, “Personal characteristics and qualities” demonstrated the highest performance, with a median score of 30 (P25–P75: 28–35) and a score rate of 85.71%. “Knowledge of NI prevention and control,” “Management and coordination ability,” and “Motivation” each showed a score rate of 80.00%, with median scores of 64 (P25–P75: 60–73), 40 (P25–P75: 37–44), and 24 (P25–P75: 22–27), respectively. “Skills in NI prevention and control” had a score rate of 78.89%, with a median score of 71 (P25–P75: 64–78). “Teaching and research ability” ranked lowest, with a score rate of 71.68% and a median score of 43 (P25–P75: 39–48). Several influencing factors were identified, including “years of experience in NI management” (*p* < 0.001, *β* = 0.133, and 95% CI = 1.574–4.989), technical title (*p* < 0.001, *β* = 0.177, and 95% CI = 3.208–22.460), and average monthly income (*p* < 0.001, *β* = 0.196, and 95% CI = 4.557–11.875).

**Conclusion:**

Competency of ICNs is at the upper‐middle level: The focus of improving the competency of ICNs is to strengthen the training of NI prevention and control skills, including NI detection, risk identification, and information construction, and to improve their teaching and research ability.

## 1. Introduction

Nosocomial infection (NI) refers to infections that patients acquire during a hospital stay. These infections do not include conditions that start before admission or are already in the incubation stage at admission [1]. In recent years, NI has become a common concern worldwide [2, 3]. In developed countries, studies report that about 3.2%–12% of hospitalized patients experience NI [4–6]. The World Health Organization (WHO) surveyed 55 hospitals across several countries and showed an average NI rate of about 8.7% [7]. The situation is more serious in some regions. In Southeast Asia and sub‐Saharan Africa, NI rates can reach very high levels, in some reports up to 75% [8]. In these regions, NI contributes to about 25%–33% of inpatient deaths [9]. In China, several cross‐sectional studies report NI rates between 0.41% and 5.46% [10–12]. NI places a heavy burden on health systems and patients [13]. Studies show that NI extends hospital stays by an average of 7.46 days per patient [14]. International studies estimate that the direct cost of one NI case ranges from 4000 to 14,570 USD [15–17]. Studies from China report costs ranging from 7100 to 73,000 RMB per case [18–21]. Large‐scale data further show the impact of NI. In the United States, the Centers for Disease Control and Prevention report about 2 million NI cases each year. These cases lead to around 80,000 deaths and up to 4 billion USD in extra costs [22]. In China, about 4 million NI cases occur every year, leading to estimated economic losses of 16–24 billion RMB [23, 24].

Many countries have established dedicated roles and teams to control NI [25]. Surveys show that these teams mainly consist of nurses, who play a key role in daily NI prevention and control. These nurses are called infection control nurses (ICNs) [26, 27]. In the United States, ICNs are often known as nurse epidemiologists [28]. In China, ICNs are registered nurses who provide education, consultation, and coordination and conduct research related to NIs [29]. These nurses need knowledge of NI prevention, clinical practice experience, and management competency.

Studies show that implementing appropriate infection control measures by ICNs can help reduce more than one‐third of infections [30]. After ICNs joined ward monitoring and management, hand hygiene compliance among healthcare workers increased from 12.6% to 55.2% [31]. During the same period, patient bacteremia rates decreased by 88.94% [32]. ICNs work closely with patients and frontline healthcare staff. They use professional knowledge and practical skills to identify infection risks. They also find problems in daily infection management and apply targeted control measures [33]. The effectiveness of NI control in hospitals is significantly influenced by the competency levels of ICNs.

However, there is a lack of comprehensive surveys on the competency levels of ICNs. The Association for Professionals in Infection Control and Epidemiology (APIC) in the United States conducted a survey [34] measuring eight core competency dimensions, and the results indicated that nearly a third of ICNs rated themselves as “Novice” in “Employee/Occupational Health,” “Cleaning, Sterilization, Disinfection,” and “Education and Research.” In contrast, a study from Korea [35] assessed 90 ICNs, based on Benner’s framework and using six categories of practical core competency standards, revealed that ICNs in Korean scored highest in infection surveillance, epidemiological investigation, and employee/occupational health. These results diverge significantly from those of the APIC study in the United States, highlighting considerable differences in competency levels and areas of weakness among ICNs across different countries. Currently, there is no specific survey addressing the competency of ICNs in China, and existing studies often focus on specific aspects of competency, such as disinfection [36], hand hygiene [37], or research skills [38]. Additionally, research has identified that various factors, including demographic and sociological variables, individual professional status, and the NI prevention and control management environment, influence the overall competency and core dimensions to varying extents [39].

Given the severe current situation of NI and the urgent need for ICNs in clinical practice, this study aims to conduct a cross‐sectional survey to assess the competency levels and influencing factors for ICNs based on the Iceberg Model of Competency, with the goal of providing insights for developing ICNs’ workforce and improving overall NI control management in hospitals.

### 1.1. Theoretical Framework

In 1973, David McClelland formally introduced the concept of “competency” to better predict employee performance and improve personnel selection [40]. Building on this work, he proposed the Iceberg Model of Competency, which conceptualizes the underlying structure of competencies required for specific job roles [39]. The model comprises six interrelated components: knowledge, skills, social role, self‐concept, traits, and motives. Knowledge refers to an individual’s understanding and mastery of relevant information, as well as the ability to apply it effectively in practice. Skills denote the capacity to perform specific tasks and translate knowledge into observable behaviors. Social role reflects the patterns of behavior and interpersonal styles shaped by an individual’s values and attitudes. Self‐concept encompasses one’s self‐perception, including internalized values, beliefs, and self‐image. Traits refer to relatively stable personality characteristics that influence consistent responses across situations. Motives represent enduring internal drives or needs that stimulate and direct behavior [39].

These elements are closely associated with job performance and can be measured reliably and improved through training. Guided by the Iceberg Model of Competency, the questionnaires used in this study included six primary domains: NI prevention and control knowledge, NI prevention and control skills, management coordination ability, teaching and research capability, personal characteristics and quality, and motivation.

## 2. Methods

This study was approved by the Ethics Review Committee of Xiang ya School of Nursing, Central South University (no: E202155). The purpose, significance, potential risks, and benefits of the study were explained before the questionnaire was issued so as to ensure that the survey subjects voluntarily participated in the study with full informed consent.

### 2.1. Study Design

**FIGURE 1:**
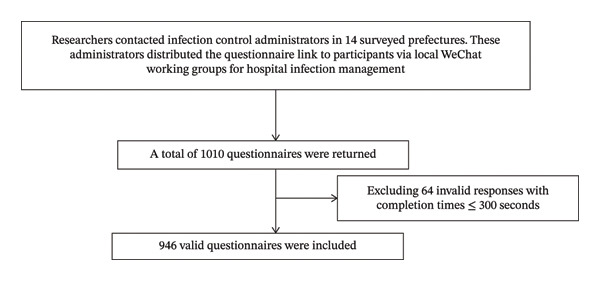
The questionnaire distribution and collection process.

This study used a convenience sampling method to conduct a cross‐sectional survey across 14 cities and states in Hunan Province, China, between December 2021 and January 2022. Out of 1010 distributed questionnaires, 64 were discarded due to an answer duration of 300 s or less, resulting in 946 valid responses and an effective recovery rate of 93.66%. Figure 1 illustrates the questionnaire distribution and collection process.

Inclusion criteria were (a) possession of a nurse practicing qualification certificate, (b) full‐time employment in a NI control department (regardless of years of service), with no part‐time clinical nursing duties; and (c) informed consent and voluntary participation. Exclusion criteria included (a) absence from work during the survey due to reasons such as sick leave, maternity leave, or study abroad and (b) status as a training nurse.

The sample size was calculated based on the following statistical formula: sample size = ${\left[U_{\alpha /2}\sigma /\delta \right]}^{2}$. For this study, *α* was set at 0.05 and *δ* was set at 0.1 times the standard deviation. From the pilot study, the standard deviation (*σ*) was determined to be 33.27. Additionally, accounting for a 10%–20% attrition rate and sampling error, the calculated sample size was 460 participants [41]. A total of 1010 ICNs finished the survey.

### 2.2. Research Tool

The questionnaire used in this study was self‐developed by the research team based on literature review, Delphi expert correspondence method, and presurvey, including two sections: general information and a competency assessment for ICNs. The general information section included 14 items on gender, age, marital status, highest education level, professional and technical title, job title, job category, employment type, years in hospital infection management, clinical nursing experience, hospital type and grade, location, whether the hospital is a teaching hospital, and average monthly income.

The competency assessment featured 69 items across 6 primary indicators: (A) Knowledge of NI prevention and control, (B) Skills in NI prevention and control, (C) Management and coordination abilities, (D) Teaching and research capabilities, (E) Personal characteristics and qualities, and (F) Motivation. Responses were scored on a Likert scale from 1 (*strongly disagree*) to 5 (*strongly agree*), with total scores ranging from 69 to 345. Higher scores indicated higher competency levels. Table 1 presents the detailed content and scoring results of each item.

**TABLE 1:** The detailed content and scoring results of each questionnaire item (*n* = 69).

Dimensions	Items	Median	P25∼P75
*A. Knowledge of NI prevention and control (n = 16)*		64	60∼73
A1. Basic knowledge (*n* = 4)		16	16∼20
	A1.1 I have a strong grasp of hospital infection‐related terms and definitions (such as hospital infection, diagnostic criteria for hospital infections, hospital infection outbreaks, suspected hospital infection outbreaks, etc.).	4	4∼5
	A1.2 I possess knowledge related to cleaning, disinfection, and sterilization.	4	4∼5
	A1.3 I am knowledgeable about standard precautions and isolation practices.	4	4∼5
	A1.4 I have a good understanding of hand hygiene for healthcare personnel.	4.5	4∼5
A2. NI pathogens, antibiotic management and other medical knowledge (*n* = 6)		22	19∼24
	A2.1 I am familiar with occupational protection knowledge (such as handling, reporting, and monitoring after occupational exposure).	4	4∼5
	A2.2 I have knowledge of common pathogens associated with hospital infections (such as types, characteristics, and treatment).	4	3∼4
	A2.3 I understand multidrug‐resistant organisms and antimicrobial management.	4	3∼4
	A2.4 I have foundational medical knowledge in microbiology, pharmacology, and immunology.	3	3∼4
	A2.5 I am knowledgeable about the epidemiology and statistics related to hospital infections.	4	3∼4
	A2.6 I am familiar with interdisciplinary knowledge in the medical field (such as medical ethics, social medicine, and health economics).	3	3∼4
A3. NI management key points and related documents (*n* = 6)		24	22∼27
	A3.1. I have a solid understanding of hospital infection management related to key departments and key sites.	4	4∼4.25
	A3.2 I have knowledge related to infection control for the three major catheter‐related infections (catheter‐related bloodstream infections, ventilator‐associated pneumonia, and catheter‐associated urinary tract infections).	4	3∼4
	A3.3 I am familiar with the hospital infection‐related regulations issued by the government, such as the “Hospital Infection Management Measures” and the “Medical Waste Management Regulations,” among others.	4	4∼4
	A3.4 I am familiar with the hospital infection‐related standards issued by the government (such as the “Standards for Hospital Infections” and the “Guidelines for Hospital Infection Surveillance”).	4	4∼4
	A3.5 I am familiar with the hospital infection‐related guidelines issued by the government (such as the “Training Guidelines for Hospital Infection Management Professionals” and the “Guidelines for the Control of Hospital Infection Outbreaks”).	4	4∼4
	A3.6 I am familiar with the regulations and policies related to hospital infections at my medical institution.	4	4∼5

*B. Skills in NI prevention and control (n = 18)*		71	64∼78
B1. Basic technology of NI prevention and control and ability of NI monitoring (*n* = 4)		16	14∼17
	B1.1 I have a strong grasp of the basic techniques for the prevention and control of hospital infections (such as hand hygiene, standard precautions, aseptic techniques, isolation techniques, and medical waste management).	4	4∼5
	B1.2 I have a solid understanding of the content and methods for hospital infection surveillance (such as monitoring hospital infection cases, monitoring the efficacy of disinfection and sterilization, environmental hygiene monitoring, monitoring the efficacy of disinfectants and medical devices, and monitoring occupational exposure among healthcare personnel).	4	4∼5
	B1.3 I can conduct monitoring tailored to the specialized characteristics of key infection control departments (such as disinfection maintenance of water treatment systems in dialysis centers, disinfection protocols in dialysis units, cleaning and disinfection processes in sterilization supply centers, and monitoring of disinfection and sterilization procedures for dental instruments).	4	3∼4
	B1.4 I am capable of collaborating with the laboratory department to conduct bacterial resistance monitoring.	3	3∼4
B2. Ability to identify NI risk and formulate prevention and control documents (*n* = 8)		31	27∼32
	B2.1 I can assess infection risks related to the layout and flow of hospital buildings, air conditioning and ventilation systems, and water use in medical institutions.	4	3∼4
	B2.2 I can evaluate infection risks associated with fabric washing and medical waste disposal.	4	4∼4
	B2.3 I can assess infection risks at key links in diagnosis, treatment, and nursing (such as anesthesia and surgery, endoscopic examinations, catheter placement, and the use of antibiotics and immunosuppressants).	4	3∼4
	B2.4 I can develop hospital infection surveillance plans based on the current situation of infections in the medical institution.	4	3∼4
	B2.5 I can formulate infection management plans for high‐risk populations, high‐risk processes, and high‐risk departments based on the objectives of hospital infection surveillance.	4	3∼4
	B2.6 I can create emergency response plans for managing hospital infection outbreaks or epidemics that are suitable for my medical institution based on national infection prevention and control regulations, standards, and guidelines.	4	3∼4
	B2.7 I can develop hospital infection prevention and control management regulations that are appropriate for the actual circumstances of the medical institution.	4	4∼4
	B2.8 I can transform experiences in hospital infection work into scientific, feasible, and promotable workflows.	4	3∼4
B3. Ability to guide and implement NI prevention and control measures (*n* = 3)		12	9∼12
	B3.1 I can guide the implementation of infection prevention and control strategies in key departments (such as intensive care units, operating rooms, neonatal wards, and burn units).	4	3∼4
	B3.2 I can guide the implementation of hospital infection control strategies for key site infections, such as surgical site infections, catheter‐related bloodstream infections, ventilator‐associated pneumonia, and catheter‐associated urinary tract infections.	4	3∼4
	B3.3 I can guide the implementation of hospital infection prevention and control strategies for multidrug‐resistant bacterial infections.	4	3∼4
B4. Building capacity of NI prevention and control informatization (*n* = 3)		11	9∼12
	B4.1 I can guide the implementation of hospital infection prevention and control strategies for multidrug‐resistant bacterial infections.	4	3∼4
	B4.2 I can analyze and interpret hospital infection data.	4	3∼4
	B4.3 I understand how to identify hospital infections or potential risks and use monitoring and diagnostic techniques to predict and analyze trends in the occurrence of hospital infections.	4	3∼4

*C. Management and coordination abilities (n = 10)*		40	37∼44
C1. Ability to organize and manage (*n* = 5)		19	16∼20
	C1.1 I can make rapid and accurate professional judgments and leadership decisions in my work.	4	3∼4
	C1.2 I have strong charisma and influence.	4	3∼4
	C1.3 I possess foresight and can anticipate trends and directions in infection control work.	4	3∼4
	C1.4 I can actively mobilize and rationally use human, material, and financial resources from medical staff, nursing, equipment, logistics, and other aspects to achieve hospital infection prevention and control goals.	4	3∼4
	C1.5 I can choose scientific, reasonable, and feasible quality improvement methods to enhance the quality of hospital infection prevention and control work.	4	3∼4
C2. Communication and collaboration ability (*n* = 5)		25	23∼30
	C2.1 I can cooperate with various levels of medical regulatory departments as a subject of inspection to complete the inspection and guidance work for my medical institution.	4	4∼4
	C2.2 I can participate as an inspector in inspections and guidance for other medical institutions in cooperation with various levels of medical regulatory departments.	4	4∼4
	C2.3 I can use communication skills for effective communication.	4	4∼4
	C2.4 I can accept constructive feedback and suggestions.	4	4∼5
	C2.5 I can provide constructive feedback and assistance to my colleagues.	4	4∼4

*D. Teaching and research capabilities (n = 12)*		4	4∼4
D1. Teaching capabilities (*n* = 4)		43	39∼48
	D1.1 I can guide and help new employees engaged in hospital infection prevention and control work with their growth and development.	16	14∼16
	D1.2 I can develop standardized and reasonable teaching/health education programs for different groups (such as doctors, nurses, interns, patients, and their families).	4	4∼4
	D1.3 I can use appropriate teaching/health education methods to impart knowledge and skills in infection control.	4	4∼4
	D1.4 I can objectively evaluate the effectiveness of teaching/health education.	4	3∼4
D2. Scientific research skills (*n* = 8)		25	23∼30
	D2.1 I can identify and propose scientific research questions that need to be addressed from the perspective of the development of the infection control discipline.	3	3∼4
	D2.2 I can retrieve literature related to hospital infection research questions and effectively analyze the literature.	3	3∼4
	D2.3 I can select appropriate research plans based on research objectives to guide the implementation of the research.	3	3∼4
	D2.4 I can adopt correct data analysis methods to process the collected research data.	3	3∼4
	D2.5 I can write standardized research reports and high‐quality papers.	3	2∼3
	D2.6 I can promote the translation of research results and apply them to hospital infection prevention and control work.	3	2∼4
	D2.7 I possess creative thinking and innovative abilities.	3	3∼4
	D2.8 I possess critical thinking and can reasonably apply existing infection control knowledge and experience for evaluation, analysis, judgment, decision‐making, and assessment.	3	3∼4

*E. Personal characteristics and qualities (n = 7)*		4	4∼4
E1. Personal traits (*n* = 4)		24	22∼27
	E1.1 I can confidently address the challenges in my work.	16	16∼18
	E1.2 I can patiently carry out hospital infection prevention and control work without haste, responding calmly to situations.	4	4∼5
	E1.3 I can maintain clarity of thought, decisiveness, and handle emergencies calmly.	4	4∼5
	E1.4 When I encounter difficulties or obstacles in my work, I can persevere to the end without easily giving up.	4	4∼5
E2. Professional values (*n* = 3)		13	12∼15
	E2.1 I believe that hospital infection prevention and control work is very necessary.	4	4∼5
	E2.2 I think my work is very important for hospital infection prevention and control.	5	4∼5
	E2.3 I understand the roles undertaken by specialized nurses in hospital infection management.	4	4∼5

*F. Motivation (n = 6)*		24	22∼27
	F1. I can find a sense of achievement and professional satisfaction in my daily work.	4	4∼4
	F2. I can promote the progress of infection control work through the sense of achievement I gain from my work.	4	4∼4
	F3. I have a clear personal career development plan.	4	3∼4
	F4. I can conduct regular self‐evaluations, comprehensively and accurately analyze my current situation, and make dynamic adjustments.	4	4∼4
	F5. I can actively learn professional knowledge and skills in hospital infection prevention and control, enhancing my knowledge and skill level.	4	4∼5
	F6. I can become an expert in the field of hospital infection prevention and control.	3	3∼4

The questionnaire demonstrated strong reliability, with a Cronbach’s *α* coefficient of 0.98, with dimension‐specific Cronbach’s *α* coefficients ranging from 0.92 to 0.97, indicating excellent internal consistency [41]. The overall content validity of the questionnaire was 0.93, while individual item content validity ranged from 0.84 to 1.00. The split‐half reliability was 0.89, indicating good reliability [41].

### 2.3. Data Collection

In November 2021, the researcher contacted 14 persons in charge of NI management in the targeted cities and states, who then shared the questionnaire link via the NI management WeChat group. Respondents filled out the questionnaire anonymously online. The questionnaire included an introduction explaining the survey’s purpose, content, and instructions. It assured respondents that the information provided would be used solely for this study and kept strictly confidential. Completing the questionnaire took approximately 5–8 min, and all questions had to be answered before submission. To prevent duplicate responses, the questionnaire settings allowed only one submission per IP address, mobile phone, or computer. Those completed in less than 300 s were considered invalid and removed.

### 2.4. Statistical Analysis

SPSS 26.0 was used to establish a database. Two individuals entered and verified the data to ensure accuracy and completeness. A bilateral test was adopted, with a statistical significance level set at *α* = 0.05. (a) Descriptive analysis: frequencies and percentages were used to describe categorical data such as age, gender, and years of work. Competency scores were expressed as median and quartile. (b) Univariate analysis: competency scores were compared based on different characteristics. For binary classification data, the Mann–Whitney *U* test was employed due to the variance was not homogeneous. For multiclassification data, the Kruskal–Wallis H test was applied for nonhomogeneous variances. (c) Multifactor analysis: multiple linear regression analysis was conducted (*α* input = 0.05 and output = 0.10). Statistical significance was determined at *p* < 0.05.

## 3. Results

### 3.1. The Demographic and Professional Information of ICNs

Table 2 presents the demographic and professional information of the survey participants. Among the 946 ICNs surveyed, 99.26% were women. The majority were aged between 31 and 40 (40.59%), followed by those aged 41–50 (36.47%). Most participants were married (89.85%), with the highest education level being undergraduate (55.81%), followed by junior college (43.76%).

**TABLE 2:** Basic information of respondents and univariate analysis of overall competency of specialized nurses in hospital infection management (*n* = 946).

Factors	Category	Participants *N* (%)	Median	P25∼P75	*H*/*Z*	*p*
Gender	Male	7 (0.74)	253.00	222.00∼345.00	−0.440^a^	0.660
	Female	939 (99.26)	263.00	240.00∼285.00		

Age (years)	≤ 30	120 (12.68)	259.50	228.00∼285.00	26.647^b^	≤ 0.001
	31∼40 (including 40)	384 (40.59)	261.00	235.00∼280.75		
	41∼50 (including 50)	345 (36.47)	262.00	242.50∼281.50		
	> 50	97 (10.25)	276.00	256.50∼304.00		

Marital status	Spinsterhood	66 (6.98)	257.00	230.50∼285.25	2.050^b^	0.359
	Married	850 (89.85)	263.00	241.00∼284.00		
	Divorced or widowed	30 (3.17)	264.00	233.50∼292.25		

Highest education	Associate degree	414 (43.76)	259.00	238.00∼276.00	8.621^b^	0.013
	Undergraduate	528 (55.81)	267.00	241.25∼289.75		
	Master degree or above	4 (0.42)	276.50	253.00∼317.25		

Technical titles	Nurse	71 (7.51)	261.00	231.00∼281.00	24.638^b^	≤ 0.001
	Senior nurse	203 (21.46)	260.00	236.00∼276.00		
	Supervisor nurse	451 (47.67)	260.00	238.00∼281.00		
	Associate chief nurse and above	209 (22.09)	272.00	251.50∼299.00		
	Other	12 (1.27)	266.50	254.25∼284.00		

Positions	No position	301 (31.82)	260.00	240.00∼287.00	17.327^b^	0.002
	Director	300 (31.71)	268.50	246.00∼287.00		
	Deputy director	54 (5.71)	271.50	247.75∼293.75		
	Head nurse	188 (19.87)	257.50	235.00∼275.00		
	Other positions	103 (10.89)	258.00	232.00∼281.00		

Job category	Technical staff	544 (57.51)	259.50	236.00∼278.75	16.125^b^	≤ 0.001
	Management staff	389 (41.12)	268.00	247.00∼288.00		
	Support staff	13 (1.37)	246.00	224.00∼273.50		

Form of employment	Officially employed	621 (65.64)	263.00	240.00∼286.00	1.260^b^	0.739
	Personnel agency	29 (3.07)	270.00	245.00∼285.00		
	Contract employment	264 (27.91)	263.00	238.00∼281.75		
	Other	32 (3.38)	258.50	239.75∼275.75		

Working experience in hospital infection management department	≤ 1 year	209 (22.09)	252.00	231.00∼275.50	39.609^b^	≤ 0.001
	1∼3 years (including 3 years)	291 (30.76)	260.00	239.00∼278.00		
	3∼5 years (including 5 years)	152 (16.07)	262.00	237.00∼286.00		
	5∼10 years (including 10 years)	163 (17.23)	269.00	250.00∼288.00		
	10∼15 years (including 15 years)	73 (7.72)	274.00	256.50∼303.50		
	> 15 years	58 (6.13)	273.50	253.75∼310.00		

Have clinical nursing experience	Yes	872 (92.18)	263.50	241.00∼285.00	−2.156^a^	0.031
	No	74 (7.82)	256.50	224.00∼275.25		

Type of hospital	Public hospital	788 (83.30)	264.00	240.00∼285.00	−0.425^a^	0.671
	Private hospital	158 (16.70)	259.50	239.75∼281.00		

Grade of hospital	Tertiary hospitals	210 (22.20)	274.50	250.00∼299.00	28.013^b^	≤ 0.001
	Second‐class hospital	487 (51.48)	262.00	242.00∼280.00		
	First‐class hospital	249 (26.32)	256.00	232.00∼276.00		

Whether it is a teaching hospital	Yes	330 (34.88)	271.00	250.00∼294.00	−5.154^a^	≤ 0.001
	No	616 (65.12)	258.50	236.00∼277.00		

Average monthly earnings (yuan)	≤ 2000	20 (2.11)	248.00	210.75∼273.25	66.892^b^	≤ 0.001
	2000∼4000 (including 4000)	353 (37.32)	257.00	231.50∼276.00		
	4000∼6000 (including 6000)	380 (40.17)	261.50	241.25∼279.00		
	6000∼9000 (including 9000)	156 (16.49)	275.00	253.25∼303.50		
	> 9000	37 (3.91)	288.00	263.50∼315.50		

^a^Represents the *Z* value obtained from the Mann–Whitney *U* test.

^b^Represents the *H* value obtained from the Kruskal–Wallis *H* test.

The technical titles were primarily those of head nurses, comprising 47.67% of the respondents. Regarding positions, nonsupervisory roles and directors were most common, each representing 31.82% and 31.71%, respectively. Professional and technical roles dominated, accounting for 57.51%, with management positions following at 41.12%. Formal employment was the primary employment type, representing 65.64%.

In terms of experience, 30.76% had worked in the NI management department for 1–3 years. A significant majority had clinical nursing experience (92.18%). Public hospitals employed the majority of participants (83.30%), with most working in secondary hospitals (51.48%) and nonteaching hospitals (65.12%). The most common average monthly income was between 4000 and 6000 (including 6000), accounting for 40.17% of the respondents.

### 3.2. Competency Scores of ICNs

**FIGURE 2:**
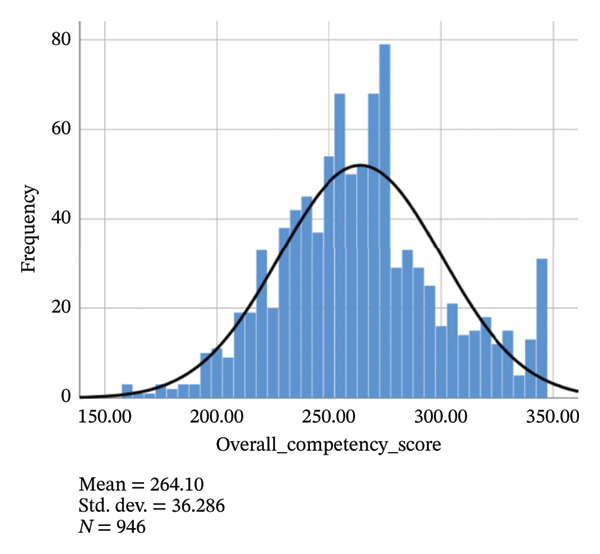
The histogram of the total competency scores.

Figure 2 illustrates the histogram of the total competency scores. Table 3 reports the specific scores of the overall competency, each primary domain, and each secondary subdomain.

**TABLE 3:** The specific scores of the overall competency, each primary domain, and each secondary subdomain (*n* = 946).

Dimensions	Items	Median	P25∼P75	Theoretical maximum total score	Scoring rate (%)
Overall competency score	69	272	253∼300	345.00	78.84
First‐level indicators	6				
Knowledge of NI prevention and control (A)	16	64	60∼73	80.00	80.00
Skills in NI prevention and control (B)	18	71	64∼78	90.00	78.89
Management and coordination abilities (C)	10	40	37∼44	50.00	80.00
Teaching and research capabilities (D)	12	43	39∼48	60.00	71.67
Personal characteristics and qualities (E)	7	30	28∼35	35.00	85.71
Motivation (F)	6	24	22∼27	30.00	80.00
Secondary competency domains	14				
Basic knowledge of hospital infection prevention and control (A1)	4	16	16∼20	20	80.00
Knowledge of hospital infection pathogens, antimicrobial stewardship, and other medical knowledge (A2)	6	22	19∼24	30	73.33
Key aspects of hospital infection management and relevant policies/documents (A3)	6	24	22∼27	30	80.00
Basic techniques for hospital infection prevention and control and surveillance capacity (B1)	4	16	14∼17	20	80.00
Ability to identify hospital infection risks and develop prevention and control protocols (B2)	8	31	27∼32	40	77.50
Ability to guide and implement hospital infection prevention and control measures (B3)	3	12	9∼12	15	80.00
Capacity for information system development in hospital infection prevention and control (B4)	3	11	9∼12	15	73.33
Organizational and management ability (C1)	5	19	16∼20	25	76.00
Communication and coordination ability (C2)	5	20	19∼22	25	80.00
Teaching ability (D1)	4	16	14∼16	20	80.00
Scientific research ability (D2)	8	25	23∼30	40	62.50
Personal traits (E1)	4	16	16∼18	20	80.00
Professional values (E2)	3	13	12∼15	15	86.67
Motivation (F)	6	24	22∼27	30	80.00

*Note:* Scoring method: each item was rated on a 5‐point Likert scale (1–5 points).

#### 3.2.1. Overall Competency Score

The competency questionnaire for ICNs had a total possible score of 345 points, with individual items scored from 1 to 5 points. The overall competency score was 272 (P25–P75: 253–300) points.

Among the six primary competency domains, when ranked from highest to lowest according to the score rate (score rate = median score/theoretical maximum total score × 100%), the results were as follows: “Personal characteristics and qualities” achieved the highest score rate of 85.71%, with a median score of 30 (P25–P75: 28–35). “Knowledge of NI prevention and control,” “Management and coordination ability,” and “Motivation” each demonstrated a score rate of 80.00%, with median scores of 64 (P25–P75: 60–73), 40 (P25–P75: 37–44), and 24 (P25–P75: 22–27), respectively. “Skills in NI prevention and control” showed a score rate of 78.89%, with a median score of 71 (P25–P75: 64–78). “Teaching and research ability” ranked the lowest, with a score rate of 71.68% and a median score of 43 (P25–P75: 39–48).

#### 3.2.2. Scores of Indicators at Each Level

a. “Knowledge of NI prevention and control” comprised three secondary indicators. Ranked by score rate from highest to lowest, A1 (basic knowledge of hospital infection prevention and control) and A3 (key aspects of hospital infection management and relevant policies/documents) both achieved a score rate of 80.00%, with median scores of 16 (P25–P75: 16–20) and 24 (P25–P75: 22–27), respectively. A2 (knowledge of hospital infection pathogens, antimicrobial stewardship, and other medical knowledge) had a lower score rate of 73.33%, with a median score of 22 (P25–P75: 19–24).
b. “Skills in NI prevention and control” included four secondary indicators. B1 (basic techniques for hospital infection prevention and control and surveillance capacity) and B3 (ability to guide and implement infection prevention and control measures) both showed a score rate of 80.00%, with median scores of 16 (P25–P75: 14–17) and 12 (P25–P75: 9–12), respectively. B2 (ability to identify hospital infection risks and develop prevention and control protocols) demonstrated a score rate of 77.50%, with a median score of 31 (P25–P75: 27–32). B4 (capacity for information system development in infection prevention and control) had a score rate of 73.33%, with a median score of 11 (P25–P75: 9–12).
c. “Management and coordination ability” consisted of two secondary indicators. The median score for C1 (organizational and management ability) was 19 (P25–P75: 16–20), corresponding to a score rate of 76.00%, while C2 (communication and coordination ability) achieved a higher median score of 20 (P25–P75: 19–22) and a score rate of 80.00%.
d. “Teaching and research ability” comprised two secondary indicators. D1 (teaching ability) had a median score of 16 (P25–P75: 14–16) and a score rate of 80.00%, whereas D2 (scientific research ability) demonstrated a markedly lower score rate of 62.50%, with a median score of 25 (P25–P75: 23–30).
e. “Personal characteristics and qualities” included two secondary indicators. E1 (personal traits) showed a median score of 16 (P25–P75: 16–18) and a score rate of 80.00%, while E2 (professional values) achieved the highest score rate within this domain at 86.67%, with a median score of 13 (P25–P75: 12–15).
f. “Motivation” did not include secondary indicators. It had a median score of 24 (P25–P75: 22–27), corresponding to a score rate of 80.00%.

### 3.3. Influencing Factors of the Competence of ICNs

#### 3.3.1. Single Factor Analysis

Univariate analysis showed that “age, highest education, technical title, job title, job category, years of work in hospital infection management department, clinical nursing work experience, hospital grade, whether the hospital was a teaching hospital, and average monthly income” had statistical significance on the overall competence of ICNs (*p* < 0.05). Gender, marital status, employment form, and hospital type had no statistical significance on the total score of the competence of ICNs.

#### 3.3.2. Multiple Linear Regression Analysis

##### 3.3.2.1. Factors Influencing Overall Competency

Variables that have a statistically significant impact on overall competency in univariate analysis were taken as independent variables, overall competency was taken as a dependent variable, and multiple linear stepwise regression analysis was performed. Table 4 lists the values of the arguments. Table 5 presents the results of the multiple linear regression analysis. The results showed that “professional title (*p* < 0.01, *β* = −0.177, and 95% CI = 3.208–22.460), average monthly income (*p* < 0.01, *β* = 0.196, and 95% CI = 4.557–11.875), and working years in hospital infection management department” (*p* < 0.01, *β* = 0.133, and 95% CI = 1.574–4.989) were the influencing factors for the overall competence of ICNs.

**TABLE 4:** Assignment table of influencing factors for competency of hospital infection control nurses.

Variable	Assignment instructions
Ages	≤ 30 = 1; 31∼40 (including 40) = 2; 41∼50 (including 50) = 3; > 50 = 4
Highest education	Use “Associate Degree” as the reference category and set dummy variables as follows: Bachelor’s Degree = (0, 1, 0); Master’s Degree and above = (0, 0, 1)
Technical titles	Use “nurse” as the reference category and set dummy variables as follows: Nurse Practitioner = (0, 1, 0, 0, 0); Charge Nurse = (0, 0, 1, 0, 0); Deputy Chief Nurse and above = (0, 0, 0, 1, 0); Other = (0, 0, 0, 0, 1)
Positions	Use “no position” as the reference category and set dummy variables as follows: Director = (0, 1, 0, 0, 0); Deputy Director = (0, 0, 1, 0, 0); Head Nurse = (0, 0, 0, 1, 0); Other = (0, 0, 0, 0, 1)
Job category	Use “professional technical position” as the reference category and set dummy variables as follows: Management position = (0, 1, 0); Operational skill position = (0, 0, 1)
Form of employment	Use “permanent staff” as the reference category and set dummy variables as follows: Personnel agency = (0, 1, 0, 0); Contract employment = (0, 0, 1, 0); Other = (0, 0, 0, 1)
Working experience in hospital infection management department	≤ 1 year = 1; 1∼3 years (including 3 years) = 2; 3∼5 years (including 5 years) = 3; 5∼10 years (including 10 years) = 4; 10∼15 years (including 15 years) = 5; > 15 years = 6
Have clinical nursing experience	Yes = 1; No = 2
Grade of hospital	Use tertiary hospitals as the reference category and set dummy variables as follows: secondary hospitals = (0, 1, 0); primary hospitals = (0, 0, 1)
Whether it is a teaching hospital	Yes = 1; No = 2
Average monthly earnings	≤ 2000 = 1; 2000∼4000 (including 4000) = 2; 4000∼6000 (including 6000) = 3; 6000∼9000 (including 9000) = 4; > 9000 = 5

**TABLE 5:** Multiple linear regression analysis of factors affecting the overall competency of hospital infection control nurses (*n* = 946).

Variables	Category	Unnormalized coefficient		Standardization coefficient	*t*	*p*	95% CI of B		Collinearity diagnostics	
		B	Standard error	*β*			Lower limit	Upper limit	Tolerance	VIF
(Constant)		242.029	6.866		35.248	≤ 0.001	228.554	255.505		

Highest education	Undergraduate	4.442	2.473	0.061	1.796	0.073	−0.411	9.296	0.850	1.177
	Master degree or above	−3.478	17.861	−0.006	−0.195	0.846	−38.531	31.574	0.954	1.048

Technical titles	Nurse	−3.662	4.906	−0.041	−0.746	0.456	−13.290	5.966	0.316	3.166
	Senior nurse	−12.834	4.905	−0.177	−2.617	0.009	−22.460	−3.208	0.214	4.684
	Supervisor nurse	−12.353	6.092	−0.141	−2.028	0.043	−24.309	−0.396	0.201	4.986
	Associate chief nurse and above	13.190	11.182	0.041	1.180	0.238	−8.755	35.135	0.818	1.222

Position	Director	−0.462	3.482	−0.006	−0.133	0.894	−7.296	6.372	0.488	2.050
	Deputy director	1.315	5.338	0.008	0.246	0.806	−9.161	11.791	0.835	1.197
	Head nurse	−1.161	3.779	−0.013	−0.307	0.759	−8.578	6.255	0.563	1.775
	Other positions	−0.874	4.199	−0.008	−0.208	0.835	−9.114	7.366	0.749	1.335

Job category	Management staff	4.527	2.586	0.061	1.751	0.080	−0.547	9.602	0.791	1.263
	Support staff	−13.067	9.976	−0.042	−1.310	0.191	−32.645	6.511	0.950	1.053

Grade of hospital	Second‐class hospital	−5.377	3.249	−0.074	−1.655	0.098	−11.754	1.000	0.486	2.058
	First‐class hospital	−6.595	4.212	−0.080	−1.566	0.118	−14.861	1.670	0.372	2.685

Average monthly earnings		8.216	1.864	0.196	4.407	≤ 0.001	4.557	11.875	0.492	2.033

Working experience in hospital infection management department		3.281	0.870	0.133	3.772	≤ 0.001	1.574	4.989	0.781	1.280

**FIGURE 3:**
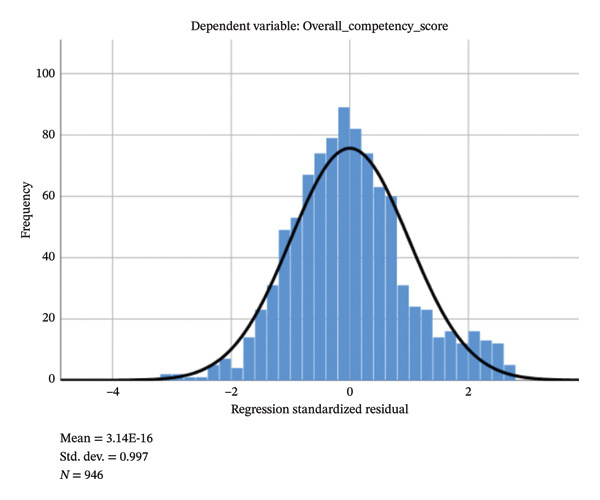
Histogram of regression standardized residuals.

**FIGURE 4:**
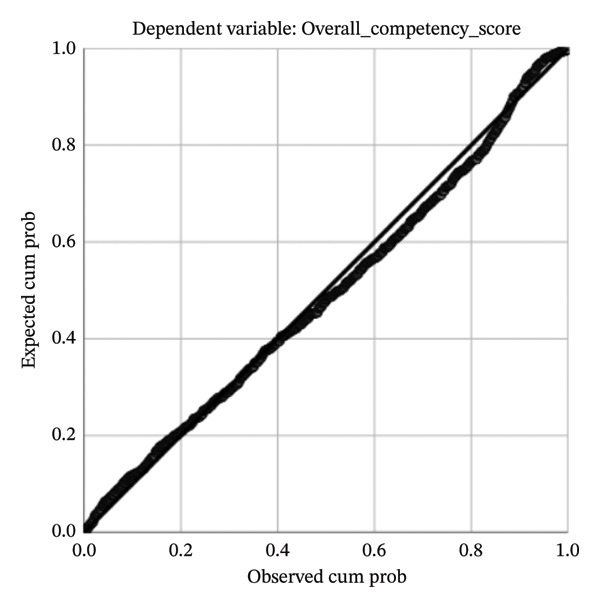
Normal P–P plot of regression standardized residual.

**FIGURE 5:**
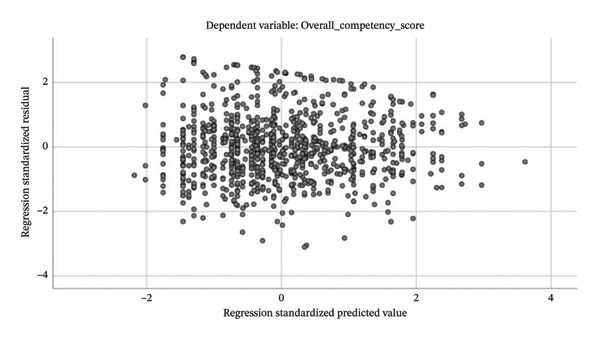
Scatterplot of standardized residuals versus standardized predicted values.

The results of the multicollinearity diagnostics indicated that the tolerance values of all independent variables ranged from 0.201 to 0.954, and the variance inflation factor (VIF) values ranged from 1.048 to 4.986. All values were within commonly accepted thresholds (tolerance > 0.10 and VIF < 10), suggesting that no serious multicollinearity existed among the independent variables. The histogram of standardized residuals (Figure 3) showed an approximately normal distribution with a mean of 0 and a standard deviation of 0.997. Together with the normal P–P plot (Figure 4) and the residual scatterplot (Figure 5), the results suggest that the assumptions of normality and homoscedasticity of residuals required for linear regression were satisfied, indicating a good overall model fit.

##### 3.3.2.2. Factors Influencing Each Dimension of Competency

Table 6 shows the results of multiple linear regression analysis of each primary domain. “Average monthly income” significantly affected all six primary indicators (*p* < 0.05). “The number of years working in the hospital infection management department” influenced “Knowledge of NI prevention and control (A), Skills in NI prevention and control (B), Management and coordination ability (C), and Teaching and research ability (D)” of ICNs (*p* < 0.05) but did not affect “Personal characteristics and motivation (E)” and “Motivation (F).” “Professional technical titles” significantly impacted “Knowledge of infection prevention and control (A), Management and coordination ability (C), Teaching and research ability (D), and Motivation (F)” (*p* < 0.05). “Whether the hospital was a teaching hospital” influenced “Skills in NI prevention and control (B)” (*p* < 0.05). “Job titles” affected “Management and coordination ability (C)” (*p* < 0.05). “The highest level of education” influenced “Teaching and research ability (D)” (*p* < 0.05). “The hospital grade” impacted “Personal characteristics and motivation (E)” and “Motivation (F)” (*p* < 0.05). “Job category” affected “Motivation (F)” (*p* < 0.05).

**TABLE 6:** Multiple linear regression analysis of each primary domain (*n* = 946).

Dimensions	Variables	Unnormalized coefficient		Standardization coefficient	*t*	*p*	95% CI of B	
		B	Standard error	*β*			Lower limit	Upper limit
(A) Knowledge of NI prevention and control	(Constant)	58.441	1.650		35.411	≤ 0.001	55.202	61.680
	Senior Nurse	−2.884	1.179	−0.166	−2.447	0.015	−5.198	−0.571
	Associate chief nurse and above	−3.057	1.464	−0.146	−2.087	0.037	−5.930	−0.183
	Years of work experience in the Hospital Infection Control Department	0.907	0.209	0.154	4.336	≤ 0.001	0.496	1.317
	Average monthly income (yuan)	1.848	0.448	0.184	4.124	≤ 0.001	0.968	2.727

(B) Skills in NI prevention and control	(Constant)	62.668	2.586		24.233	≤ 0.001	57.593	67.743
	Average monthly income (yuan)	2.308	0.589	0.173	3.917	≤ 0.001	1.152	3.464
	Whether it is a teaching hospital	−2.378	0.973	−0.098	−2.444	0.015	−4.288	−0.469
	Years of work experience in the Hospital Infection Control Department	1.248	0.273	0.159	4.574	≤ 0.001	0.712	1.783

(C) Management and coordination ability	(Constant)	34.266	1.062		32.262	≤ 0.001	32.181	36.350
	Senior nurse	−1.894	0.759	−0.168	−2.497	0.013	−3.383	−0.405
	Director	1.367	0.539	0.113	2.539	0.011	0.310	2.425
	Average monthly income (yuan)	1.308	0.288	0.201	4.535	≤ 0.001	0.742	1.874
	Years of work experience in the Hospital Infection Control Department	0.425	0.135	0.111	3.157	0.002	0.161	0.689

(D) Teaching and research ability	(Constant)	42.080	1.870		22.506	≤ 0.001	38.411	45.750
	Undergraduate	1.233	0.590	0.077	2.092	0.037	0.076	2.390
	Senior nurse	−4.034	1.169	−0.253	−3.452	0.001	−6.328	−1.741
	Associate chief nurse and above	−3.607	1.462	−0.188	−2.468	0.014	−6.476	−0.739
	Years of work experience in the Hospital Infection Control Department	0.529	0.199	0.098	2.657	0.008	0.138	0.920
	Average monthly income (yuan)	0.865	0.420	0.094	2.061	0.040	0.041	1.689

(E) Personal characteristics and motivation	(Constant)	27.510	0.662		41.527	≤ 0.001	26.210	28.810
	Secondary hospital	−0.902	0.305	−0.128	−2.958	0.003	−1.500	−0.303
	Average monthly income (yuan)	0.857	0.177	0.211	4.846	≤ 0.001	0.510	1.204

(F) Motivation	(Constant)	22.808	0.724		31.506	≤ 0.001	21.387	24.228
	Senior nurse	−1.274	0.505	−0.164	−2.520	0.012	−2.266	−0.282
	Associate chief nurse and above	−1.513	0.609	−0.162	−2.486	0.013	−2.708	−0.319
	Technical and support staff position	−2.737	1.082	−0.082	−2.529	0.012	−4.861	−0.613
	Secondary hospital	−1.208	0.331	−0.155	−3.653	≤ 0.001	−1.858	−0.559
	Primary hospital	−0.831	0.400	−0.094	−2.076	0.038	−1.617	−0.046
	Average monthly income (yuan)	0.753	0.194	0.168	3.887	≤ 0.001	0.373	1.132

*Note:* Only statistically significant variable results are shown.

## 4. Discussion

### 4.1. Current Status of ICNs Overall Competency

A cross‐sectional survey using a self‐designed competency questionnaire for ICNs was conducted in 14 cities and states in Hunan Province. The questionnaire measured six core indicators of the competency of ICNs: Knowledge of NI prevention and control (A), Skills in NI prevention and control (B), Management and coordination abilities (C), Teaching and research capabilities (D), Personal characteristics and qualities (E), and Motivation (F). The results revealed that the overall competency score was 272 (P25–P75: 253–300) points and the scoring rate was 78.84%. Considering the total possible score range of 69–345 and an item score range of 1–5, the competency level of ICNs was above the medium level, indicating that ICNs are relatively capable of fulfilling their responsibilities. However, there were statistically significant differences among the six primary indicators.

### 4.2. Current Status of ICNs Competency Across Dimensions

Domain D (Teaching and research ability) showed the lowest score rate (71.68%). This domain comprised two secondary indicators. D2 (scientific research ability) had a score rate of 62.50%, ranking the lowest among all secondary indicators, whereas D1 (teaching ability) achieved a score rate of 80.00%. These results suggest that teaching and research competencies warrant particular attention, especially research capacity. Currently, nursing education focuses primarily on professional knowledge and skills, often neglecting the development of research capabilities [42]. Additionally, ICNs have limited access to research knowledge, typically through hospital‐organized lectures without comprehensive research training, leading to a lack of understanding or application of research knowledge [43]. Therefore, there is a need to enhance the scientific and academic research levels of ICNs [44].

The second lowest score rate was observed in Domain B (Skills in NI prevention and control) at 78.89%. This domain included four secondary indicators. B4 (capacity for information system development in infection prevention and control) had the lowest score rate (73.33%), followed by B2 (ability to identify hospital infection risks and develop prevention and control protocols) with a score rate of 77.50%. In contrast, B1 (basic techniques for infection prevention and control and surveillance capacity) and B3 (ability to guide and implement infection prevention and control measures) both achieved score rates of 80.00%, aligning with Chen Huisi et al.’s [39] evaluation of the competency of infection control personnel across various medical institutions in Jilin Province. Information technology construction is a crucial step in NI prevention and control, directly impacting the quality of NI control efforts [45, 46]. However, research indicates that in some medical institutions, the networked and digitized management of NI lags significantly. Case monitoring often relies on retrospective reviews of medical records by ICNs, which is time‐consuming and labor‐intensive, compromising the scientific accuracy and timeliness of the data collected [47]. It is essential to provide comprehensive training for ICNs in areas such as monitoring, document formulation, and the construction of information technology systems. This can be achieved through continuing education, academic conferences, emergency drills, or further training, thereby facilitating the learning and updating of NI control management skills [46].

The highest score rate among the six domains was observed in Domain E (Personal characteristics and qualities) at 85.71%. This domain comprised two secondary indicators, with E2 (professional values) achieving a score rate of 86.67% and E1 (personal traits) a score rate of 80.00%. While personal traits and qualities are often implicit and easily overlooked compared with knowledge and skills, they significantly impact the long‐term professional development of ICNs [48]. The self‐concept, values, and personality traits of ICNs should be developed in tandem with their knowledge and skills, with enhanced educational efforts in these areas to stimulate their intrinsic motivation [39, 49, 50]. Previous study has shown the work engagement of Chinese ICNs is at a relatively high level [51]. ICNs possess a robust intrinsic motivation to make positive adjustments in their work, which is a crucial factor in maintaining their competency levels [39, 52].

The remaining three domains demonstrated identical score rates of 80.00%. Within Domain A (Knowledge of NI prevention and control), which included three secondary indicators, A2 (knowledge of hospital infection pathogens, antimicrobial stewardship, and other medical knowledge) showed a lower score rate (73.33%) compared with A1 (basic knowledge of hospital infection prevention and control) and A3 (key aspects of hospital infection management and relevant policies/documents), indicating that ICNs’ mastery of specialized infection prevention and control knowledge remains insufficient and requires further strengthening. Han Lingyang et al. [44] similarly noted that nursing staff excel in disinfection and isolation but have relatively weak knowledge in the guidance of antimicrobial use and the management of multidrug‐resistant organisms. It is imperative that ICNs possess a robust foundation in NI prevention and control, particularly in the epidemiological characteristics of common pathogens and antimicrobial management, as these are focal points of the discipline.

Within Domain C (Management and coordination ability), the score rate for organizational and management ability (C1) (76.00%) was lower than that for communication and coordination ability (C2) (80.00%), aligning with the findings of Chen Huisi’s study [39]. Currently, due to the differences in job responsibilities, head nurses or those in administrative positions at the director level or above are required to have strong management skills, while ordinary nurses primarily focus on performing their duties well [53]. In a study where ICNs were jointly led by the NI control department and the nursing department, and they were also given certain management authorities and responsibilities, it was found that this approach facilitated more effective NI control work [54]. The NI management department, which functions both as an administrative office and a business unit, has a complex scope of work that spans across the hospital. Collaboration across the entire hospital is fundamental for the successful implementation of NI management, thus placing higher demands on the management and collaboration capabilities of ICNs [54].

### 4.3. Analysis of Factors Influencing Competency of ICNs

The findings indicated that longer tenure in the infection control department was associated with higher overall competency among ICNs (*p* < 0.001). Moreover, ICNs holding intermediate professional titles demonstrated significantly higher competency levels than those with primary titles (*p* < 0.01). Competency development is inherently cumulative and evolves through sustained professional practice. As years of service and practical experience increase, ICNs are exposed to more complex clinical scenarios, which facilitates the integration of theoretical knowledge with practical skills. This trajectory aligns with Benner’s “Novice to Expert” framework [55], which conceptualizes professional growth as a progression grounded in experiential learning. Conversely, newly appointed ICNs may exhibit lower competency levels due to limited practical experience, unfamiliarity with institutional systems, and insufficient role clarity. These challenges may also reflect gaps in structured career development pathways. Targeted and standardized training programs are, therefore, essential to accelerate competency acquisition. Healthcare institutions should implement stratified management approaches, evaluating ICNs according to tenure and demonstrated capability, with particular emphasis on core infection prevention knowledge, technical skills, and role‐specific responsibilities [56, 57]. Significant differences in educational background and competency levels were also observed, underscoring the need for standardized qualification and certification systems for ICNs [46]. Collaborative efforts among governmental agencies, academic institutions, and professional organizations are warranted to establish clear certification criteria, including educational requirements, professional ranking, and minimum years of experience [39].

In addition, higher average monthly income was positively associated with overall competency (*p* < 0.001), consistent with prior research [58]. Current investment in NI control in China remains relatively limited, contributing to high workloads and comparatively modest compensation for ICNs. This imbalance between effort and reward may adversely affect workforce stability and professional motivation [59]. Similar concerns have been reported in Poland, where infection prevention and control roles are perceived as undervalued by healthcare staff and hospital administrators [60]. Developing equitable performance evaluation systems that incorporate competency‐based assessment and link financial incentives to demonstrated performance may support workforce retention and strengthen the development of infection control expertise [56, 57].

In addition, ICNs with bachelor’s degrees scored higher in teaching and research abilities compared with those with associate degrees (*p* < 0.01). Previous studies have shown that the weakest link in the training of associate degree nurses is research ability, which is a key aspect of competency development in the new era [61]. Strengthening cooperation between government health departments and universities, focusing on competency and quality education, and improving curriculum design to meet the competency requirements of ICNs are crucial [62, 63].

ICNs serving in director‐level roles demonstrated significantly stronger management and collaboration competencies than those without administrative positions (*p* < 0.05). This likely reflects the functional nature of infection control departments, which operate across multiple clinical units and administrative processes, so that effective management and coordination across the hospital‐wide are central to implement infection control strategies [64]. Furthermore, ICNs in technical positions scored lower in motivation compared with those in professional technical positions (*p* < 0.01). Technical positions often involve logistics support and material management, leading to lower job satisfaction and professional fulfillment [65]. This issue underscores the importance of establishing a comprehensive performance management and promotion system for ICNs.

At the organizational level, ICNs employed in tertiary hospitals score higher in “personal characteristics and motivation” compared with those working in secondary hospitals (*p* < 0.05). Tertiary hospitals generally offer more competitive compensation, stronger academic environments, and clearer career development pathways. Such institutional advantages may enhance professional identity, perceived career prospects, and confidence in one’s competencies, thereby strengthening motivational and personal development dimension [66]. Furthermore, ICNs in nonteaching hospitals scored lower in infection prevention and control skills compared with those in teaching hospitals (*p* < 0.01). Nonteaching hospitals often lack sufficient teaching resources and investment in skill training, and their faculty strength may not meet teaching demands, affecting skill practice levels [67]. Strengthening long‐term training strategies and implementing systematic goal‐oriented management may, therefore, be critical for enhancing infection control capacity in nonteaching institutions [39].

### 4.4. Limitations

This study has several limitations. Due to constraints in time, resources, and the limited number of ICNs in some institutions, convenience sampling was adopted, which may have introduced selection bias and limited the generalizability of the findings. In addition, competency was assessed using a self‐report questionnaire, making the results susceptible to response biases such as social desirability and overestimation of competence. Third, the operational definition of ICNs was relatively broad. The study participants were full‐time nurses working in hospital infection management departments; however, no strict distinction was made regarding whether they held administrative or managerial positions. In practice, nurses with administrative roles (e.g., head nurses or department directors) may differ substantially from nonadministrative ICNs who primarily focus on frontline infection prevention and control activities in terms of role responsibilities, work priorities, and competency requirements. Future research is, therefore, recommended to more clearly delineate the roles of study participants to enhance the applicability and interpretability of findings for different target groups. Finally, the competency questionnaire was developed without in‐depth qualitative interviews, which may have resulted in incomplete item coverage.

## 5. Conclusion

The competency level of ICNs is above the medium level, indicating they are relatively capable of fulfilling their duties. Factors influencing competency include years of service in the NI control department, technical title, average monthly income, highest educational level, hospital grade, whether the hospital is a teaching hospital, position, and job category. Future efforts to enhance the competency of ICNs should focus on strengthening training in NI detection, risk identification, and information system development and improving teaching and research abilities.

**Nomenclature**
NI: Nosocomial infection
ICNs: Infection control nurses

## Author Contributions

Wan Li was responsible for (a) the conception and design of the study, acquisition of data, analysis, and interpretation of data and (b) final approval of the version to be submitted. Pan Lin participated in the design of the questionnaire, analysis, drafting the article, and revising it critically for important intellectual content. Qiuhong Yang, Haifan Yang, Xin Tan, Xinyu Feng, Hui Luo, and Weijuan Li assisted in the preparation of the questionnaire. Yinglan Li, Ying Zhang, and Lingyun Tian conceptualized and designed the research, coordinated and supervised data collection, and were responsible for final approval of the version to be submitted.

## Funding

The study was supported by grant from the Youth Fund of National Natural Science Foundation of China (Grant no. 72304261).

## Disclosure

All authors have approved the final manuscript to be submitted and agreed to be accountable for all aspects of the work.

## Conflicts of Interest

The authors declare no conflicts of interest.

## Data Availability

The data that support the findings of this study are available on request from the corresponding authors. The data are not publicly available due to privacy or ethical restrictions.

## References

[1] National Health Commission of the People’s Republic of China , Guidelines for the Control of Hospital Infection Outbreaks, WS/T 524—2016, August 2, 2016, 2016.

[2] Wang Q. , Research Progress on the Status of Hospital Infection Prevention and Control Measures and Intervention Effects, China Health Standard Management. (2019) 10, no. 10, 82–85.

[3] Wen J. Q. and Shang L. P. , Current Status and Implications of Certification Systems for Infection Control Specialist Nurses at Home and Abroad, Chinese Journal of Practical Nursing. (2024) 40, no. 22, 1756–1761.

[4] Wang Y. , Overview of Hospital Infection Prevention and Control Work in China, Chinese Nursing Management. (2008) 8, no. 1, 10–12.

[5] Shelley S. M. , Erin O. L. , Sarah J. J. et al., Changes in Prevalence of Health care-associated Infections in U.S. Hospitals, New England Journal of Medicine. (2018) 379, no. 18, 1732–1744. 30380384 10.1056/NEJMoa1801550 PMC7978499

[6] Jessica L. F. , Athman M. , Yewande H. A. et al., Healthcare-Associated Outbreaks of Bacterial Infections in Africa, 2009–2018: a Review, International Journal of Infectious Diseases. (2021) 103, 469–477. 33333248 10.1016/j.ijid.2020.12.030

[7] World Health Organization , The Burden of Health care-associated Infection Worldwide [Report], 2016, World Health Organization, Geneva.

[8] Oluwagbemiga A. O. , Akinsete S. J. , and Ana G. R. , Building Conditions and the Risk of Nosocomial Infection from Microbial Contamination of Hospital Appliances in a Health Care Facility, International Journal of Environmental Health Research. (2017) 27, no. 4, 264–275, 10.1080/09603123.2017.1332350, 2-s2.0-85019759422. 28553878

[9] Xian B. S. and Fan Y. C. , A Study on the Direct Economic Loss of hospital-acquired Infections Among Inpatients in a Comprehensive Hospital in Inner Mongolia, China Health Resources. (2018) 21, no. 3, 232–234.

[10] Liu X. , Xie M. , Sun J. et al., A National Survey on the Current Status of Post Competency of full-time healthcare-associated Infection Management Personnel in 31 Provinces of China, Chinese Journal of Infection Control. (2025) 24, no. 3, 354–360.

[11] Ni L. , Du Q. , Bian H. , Ab Manan N. , and Mohamad A. R. , Current State of Training Needs and Programs for Infection Control Liaison Nurses, Frontiers of Medicine. (2025) 12, 10.3389/fmed.2025.1703523. PMC12753412 41480540

[12] Ma L. , Zhu S. , and Zhang J. , Survey on the Prevalence of hospital-acquired Infections on Survey Days from 2018 to 2020 in a Secondary County Hospital of Traditional Chinese Medicine, Medical Diet and Health. (2021) 19, no. 18, 221–223.

[13] Kang L. , Zhao H. , Yang X. et al., Study on the Role of the Hospital Infection Control Team in Improving the Cleanliness of Environmental Surfaces, Chinese Journal of Nosocomiology. (2017) 27, no. 03, 695–698.

[14] Cheng Z. P. , Chen Y. B. , Zhou Y. et al., Analysis of the Direct Economic Loss from Acinetobacter baumannii hospital-acquired Infections from 2016 to 2020 in a Hospital, Chinese Journal of Nosocomiology. (2021) 31, no. 16, 2551–2555.

[15] Heidar H. F. , Degheili J. A. , Yacoubian A. A. et al., Management of Urinary Tract Infection in Women: a Practical Approach for Everyday Practice, Urology Annals. (2019) 11, no. 4, 339–346. 31649450 10.4103/UA.UA_104_19 PMC6798292

[16] Manoukian S. , Steward S. , Graves N. , Mason H. , Robertson C. , Kennedy S. , Pan J. , Haahr L. , Dancer S. , Cook B. , and Reilly J. , Evaluating the Post-discharge Cost of healthcare-associated Infection in NHS Scotland, Journal of Hospital Infection. (2021) 114, 51–58, 10.1016/j.jhin.2020.12.026. 34301396

[17] Evans O. , Ama P. F. , Felix A. A. et al., Cost-Effectiveness Analysis of an Active 30-day Surgical Site Infection Surveillance at a Tertiary Hospital in Ghana: Evidence from HAI-Ghana Study, BMJ Open. (2022) 12, no. 1. 10.1136/bmjopen-2021-057468 PMC8724807 34980632

[18] Liu W. P. , Hai Y. T. , Xing H. M. et al., Study on the Economic Loss of hospital-acquired Infections in 24 Tertiary General Hospitals in Inner Mongolia, Chinese Journal of Disinfection. (2020) 37, no. 09, 674–678.

[19] Yang L. , Xu L. J. , and Zhang Y. Q. , Study on Risk Factors and Direct Economic Losses of hospital-acquired Infections in Orthopedic Surgery Patients, Journal of Traditional Chinese Medicine Management. (2020) 28, no. 16, 133–135.

[20] Chen Y. K. , Ji X. F. , Yu L. N. et al., Study on the Direct Economic Loss of Acinetobacter baumannii Infections in a Tertiary Hospital in 2016, Chinese Journal of Nosocomiology. (2019) 29, no. 18, 2754–2758+2762.

[21] Wang J. , Gao N. , Liu H. P. et al., A case-control Study on the Direct Economic Loss of hospital-acquired Infections in a Tertiary Pediatric Hospital, Chinese Journal of Nosocomiology. (2019) 29, no. 10, 1579–1583.

[22] Askarian M. and Gooran N. R. , National Nosocomial Infection Surveillance System-based Study in Iran: Additional Hospital Stay Attributable to Nosocomial Infections, American Journal of Infection Control. (2003) 31, no. 8, 465–468, 10.1016/s0196-6553(03)00673-4, 2-s2.0-0347383716. 14647108

[23] Sun Z. , Liu J. , and Cai M. , Literature Analysis of Hospital infection-related Research in Ten Core Nursing Journals in China from 2008 to 2014, Chinese Nursing Management. (2015) 15, no. 6, 717–720.

[24] Yu G. , Sun D. , Li Y. et al., Study on the Economic Losses of hospital-acquired Infections in China, Medicine in Society. (2016) 29, no. 07, 70–72.

[25] Dekker M. , Jongerden I. P. , Caris M. G. , de Bruijne M. C. , Vandenbroucke-Grauls C. M. J. E. , and van Mansfeld R. , Evaluation of an Infection Control Link Nurse Program: an Analysis Using the RE-AIM Framework, BMC Health Services Research. (2023) 23, no. 1, 10.1186/s12913-023-09111-5. PMC9912654 36759832

[26] Yang T. , Liu W. , Chen L. et al., Survey on the Status of Professional Settings for Hospital Infection Management in Guizhou Province, Chinese Journal of Nosocomiology. (2018) no. 8, 1249–1252.

[27] Massaroli A. , Martini J. G. , Moya L. M. , Pereira M. S. , Tipple A. F. V. , and Maestri E. , Skills for Generalist and Specialist Nurses Working in the Prevention and Control of Infections in Brazil, Rev Lat Am Enfermagem. (2019) 27, 10.1590/1518-8345.2620.3134, 2-s2.0-85065466941. PMC6528634 31038628

[28] Henman L. J. , Corrigan R. , Carrico R. , Suh K. N. , Anderson K. , Boukidjian R. , Callery S. , Conner J. , Fugate M. , Garcia-Houchins S. , Gibbons J. , Kaiser M. T. , Rebmann T. , Round L. , Rohrbach P. , Rhodenizer-Rose S. , Andrews J. , Callery S. , Conner J. , Flinchum A. , Fugate M. , Fulton M. , Hsu V. , and Smyer J. , Identifying Changes in the Role of the Infection Preventionist Through the 2014 Practice Analysis Study Conducted by the Certification Board of Infection Control and Epidemiology, Inc, American Journal of Infection Control. (2015) 43, no. 7, 664–668, 10.1016/j.ajic.2015.02.026, 2-s2.0-84937519382. 25858308

[29] Li Q. , Research on the Construction of the Training Model Index System for Specialist Nurses in Hospital Infection Control [Dissertation], 2018, Shanxi Medical University.

[30] Li X. and Dang S. , The Impact of Establishing Infection Control Monitoring Nurses on the Quality of Hospital Infection Management, China Modern Doctor. (2021) 59, no. 09, 159–162.

[31] Noh E. Y. , Lee M. H. , Yi Y. M. , and Park Y. H. , Implementation of a Multimodal Infection Control Strategy in the Nursing Home, Geriatric Nursing. (2021) 42, no. 3, 767–771, 10.1016/j.gerinurse.2021.03.020. 33895498

[32] Garvey M. I. , Bradley C. W. , Wilkinson A. C. , Holden K. L. , Clewer V. , and Holden E. , The Value of the Infection Prevention and Control Nurse Led MRSA Ward Round, Antimicrobial Resistance and Infection Control. (2019) 8, no. 1, 10.1186/s13756-019-0506-6, 2-s2.0-85062951798. PMC6417022 30911379

[33] Ma L. , Effectiveness of Hospital Infection Management Practice Based on Infection Management Nurses, Journal of Clinical Medical Literature. (2020) 7, no. 03.

[34] Kathleen A. G. , Carole L. , Raya K. et al., Advancing the Competency of Infection Preventionists, American Journal of Infection Control. (2015) 43, no. 4, 370–379. 25721061 10.1016/j.ajic.2015.01.005

[35] Kim K. M. and Choi J. S. , Self-Perceived Competency of Infection Control Nurses Based on Benner’s Framework: a Nationwide Survey in Korea, Applied Nursing Research. (2015) 28, no. 2, 175–179, 10.1016/j.apnr.2014.09.010, 2-s2.0-84928006478. 25315139

[36] Liu R. , Wang X. , Pang S. et al., Knowledge, Attitudes, and Practices Regarding Disinfection Among Hospital Infection Management Staff and Influencing Factors, Chinese Journal of Infection Control. (2021) 20, no. 08, 759–762.

[37] Yang Y. , Zhang H. , Hu L. et al., Survey on Hand Hygiene Cognition Among Hospital Infection Management Staff in Gansu Province, Chinese Journal of Infection Control. (2019) 18, no. 02, 142–146.

[38] Shi L. and Li Y. , Survey on the Status and Research Capabilities of Hospital Infection Professionals in Jiangsu Province, Jiangsu Health Care Management. (2021) 32, no. 10, 1272–1275.

[39] Chen H. , Construction and Empirical Study of the Competency Evaluation Model for Hospital Infection Prevention and Control Personnel [Dissertation], 2019, Jilin University.

[40] McClelland D. C. , Testing for Competence Rather than for Intelligence, American Psychologist. (1973) 28, 1–14, 10.1037/h0034092, 2-s2.0-0015535482. 4684069

[41] Wu M. , Practical Analysis of Questionnaire Statistics [Monograph], 2010, Chongqing University Press, Chongqing.

[42] Wu Q. Y. , Liao T. , Gao M. H. et al., Analysis of the Current Situation and Influencing Factors of Scientific Research Ability of Clinical Specialist Nurses in Sichuan Province, Journal of Nurses Training. (2021) 36, no. 07, 646–649.

[43] Ge Y. R. , Yan S. Y. , Zhao L. L. et al., Application of Action Research in Training Scientific Research Abilities of Nurses, Chinese Journal of Nursing. (2016) 51, no. 1, 75–79.

[44] Han L. Y. , Wang G. F. , Huang X. Q. et al., Analysis of Hospital Infection Management Organization Structures in 320 Hospitals, Chinese Journal of Nosocomiology. (2020) 30, no. 11, 1749–1752.

[45] Chen T. T. , Application Status and Development Trends of Hospital Infection Management Information Systems, Smart Health. (2019) 5, no. 35, 4–5.

[46] Cao J. G. , Innovative Construction and Expansion of Work Capacity in Hospital Infection Management, Chinese Journal of Nosocomiology. (2017) 27, no. 14, 3135–3138.

[47] Yi S. H. , Wang M. R. , Chen B. Z. et al., Investigation on the Status of Hospital Infection Management in 42 Traditional Chinese Medicine Hospitals in Fujian Province, Chinese Journal of Infection Control. (2018) 17, no. 04, 320–324.

[48] Lai X. X. , Li Z. , Bo L. et al., Construction of a Competency Evaluation Index System for Specialist Nurses, Modern Clinical Nursing. (2019) 18, no. 05, 68–73.

[49] Zhang Q. , Content Effects in Trait Reasoning and the Influence of Gender Stereotypes, 2012, Shandong Normal University.

[50] Rebeno D. R. , Tiongco D. D. , and Macindo J. R. B. , A Structural Equation Model on the Attributes of a Skills Enhancement Program Affecting the Clinical Competence of Pre-graduate Nursing Students, Nurse Education Today. (2017) 49, 180–186. 27988466 10.1016/j.nedt.2016.11.030

[51] Tian L. , Wu A. , Li W. et al., Relationships Between Perceived Organizational Support, Psychological Capital, and Work Engagement Among Chinese Infection Control Nurses, Risk Management and Healthcare Policy. (2023) 16, 551–562, 10.2147/rmhp.s395918. 37035271 PMC10081527

[52] Wang Y. , Su Y. , and Wang X. L. , Analysis of Competency Evaluation Indicators for Nursing Students and New Nurses, General Nursing. (2020) 18, no. 32, 4385–4390.

[53] Li Q. W. and Shang L. P. , Construction of a Training Model for Specialist Nurses in Hospital Infection Control, Journal of Nursing Science. (2018) 33, no. 10, 76–80.

[54] Yao J. , Chen X. J. , and Xing H. , Whole-Process Management of Hospital Infections Based on New Media, Chinese Journal of Infection Control. (2018) 17, no. 11, 989–992.

[55] Gobet F. and Chassy P. , Towards an Alternative to Benner’s Theory of Expert Intuition in Nursing: a Discussion Paper, International Journal of Nursing Studies. (2008) 45, no. 1, 129–139, 10.1016/j.ijnurstu.2007.01.005, 2-s2.0-36549054163. 17337269

[56] Qu X. and Yao Y. , Discussion on the Current Situation and Prevention Strategies of Hospital Infection Management, China Health Industry. (2018) 15, no. 23, 53–54.

[57] Li X. , Analysis of the Current Situation and Influencing Factors of the Competency of ICU Nurses in Tertiary Hospitals in Jilin Province [Dissertation], 2016, Changchun University of Traditional Chinese Medicine.

[58] Jia W. , Li Y. , and Ma Y. , Self-Assessment of the Competency of Infection Control Personnel in Secondary and Higher Hospitals in Xinjiang and Its Influencing Factors, China Medical Herald. (2021) 18, no. 26, 46–49.

[59] Zhang P. , Tang L. , Qian L. et al., Analysis of Job Burnout and effort-reward Imbalance Among full-time Hospital Infection Management Staff in Wuhu City, Chinese Journal of Infection Control. (2019) 18, no. 12, 1159–1164.

[60] Jaślan D. , Rosiński J. , Wałaszek M. et al., Polish Infection Control Nurses’ Job Satisfaction and Cooperation with Their Colleagues Reflect How the Value of Infection Control is Appreciated by Other Healthcare Workers: Findings from Surveys Conducted Before and During the COVID-19 Pandemic, Antimicrobial Resistance and Infection Control. (2023) 12, no. 1, 10.1186/s13756-023-01284-2. PMC10413731 37559154

[61] Cheng Y. and Shao Y. , Thoughts on the Cultivation of Nurse Competency, Chinese Nursing Education. (2018) 15, no. 04, 245–249.

[62] Li L. , New Technologies and Advancements in Hospital Infection Prevention and Control, West China Medical Journal. (2018) 33, no. 03, 240–243.

[63] Feng H. , Li G. , Xu C. et al., Training and Utilization of Specialist Nurses Based on Job Competency, Chinese Nursing Education. (2020) 17, no. 08, 681–684.

[64] Zhang R. , Shang L. P. , Su D. X. et al., Construction of a Core Competency Evaluation Index System for full-time Infection Management Personnel Using the Delphi Method, Chinese Journal of Nosocomiology. (2017) 27, no. 10, 2382–2385.

[65] Zhang J. , Gu X. , and Li Z. , Talent Cultivation and Performance Management Strategies for Technical Positions in Hospital Logistics, Science and Technology Information. (2021) 19, no. 02, 112–114.

[66] Guan J. , Comprehensive Evaluation of Infectious Disease Prevention and Control Capabilities of Medical Institutions in Xicheng District, Beijing, Journal of Preventive Medicine Information. (2018) 34, no. 04, 440–444.

[67] Liu H. , Guo R. , and Wang Y. , Study on the Current Situation and Countermeasures of Teaching Skills Training for Young Medical Physicians, Continuing Medical Education. (2021) 35, no. 03, 40–42.

